# Contrast and stability of the axon diameter index from microstructure imaging with diffusion MRI

**DOI:** 10.1002/mrm.24501

**Published:** 2012-09-28

**Authors:** Tim B Dyrby, Lise V S⊘gaard, Matt G Hall, Maurice Ptito, Daniel C Alexander

**Affiliations:** 1 Danish Research Centre for Magnetic Resonance, Copenhagen University Hospital HvidovreHvidovre, Denmark; 2 Centre for Medical Image Computing, University College LondonLondon, UK; 3 School of Optometry, University of MontrealMontreal, Canada; 4 Department of Neuroscience and Pharmacology, University of CopenhagenDenmark

**Keywords:** brain, magnetic resonance imaging, model, ActiveAx, AxCaliber, DTI, monkey

## Abstract

The ActiveAx technique fits the minimal model of white matter diffusion to diffusion MRI data acquired using optimized protocols that provide orientationally invariant indices of axon diameter and density. We investigated how limitations of the available maximal gradient strength (*G*_max_) on a scanner influence the sensitivity to a range of axon diameters. Multishell high-angular-diffusion-imaging (HARDI) protocols for *G*_max_ of 60, 140, 200, and 300 mT/m were optimized for the pulsed-gradient-spin-echo (PGSE) sequence. Data were acquired on a fixed monkey brain and Monte-Carlo simulations supported the results. Increasing *G*_max_ reduces within-voxel variation of the axon diameter index and improves contrast beyond what is achievable with higher signal-to-noise ratio. Simulations reveal an upper bound on the axon diameter (∼10 μm) that pulsed-gradient-spin-echo measurements are sensitive to, due to a trade-off between short *T*_2_ and the long diffusion time needed to probe larger axon diameters. A lower bound (∼2.5 μm) slightly dependent on *G*_max_ was evident, below which axon diameters are identifiable as small, but impossible to differentiate. These results emphasize the key-role of *G*_max_ for enhancing contrast between axon diameter distributions and are, therefore, relevant in general for microstructure imaging methods and highlight the need for increased *G*_max_ on future commercial systems.

## Introduction

A variety of geometrical models that relate the diffusion MRI signal directly to white matter microstructural features, such as axon diameter and fiber density, have been proposed 1–6. For example, Stanisz et al. 5 proposed a model with three compartments, axonal, glial, and extracellular space, separated by semipermeable membranes and with unique shapes, dimensions, volume fractions, and internal diffusivity. The AxCaliber model 2 has impermeable cylindrical axons with a gamma distribution of radii and an extracellular compartment exhibiting hindered diffusion modeled by a diffusion tensor. Barazany et al. 3 added a cerebrospinal fluid compartment for in vivo applications. Alexander et al. 1 combined elements of these earlier models, motivated by identifying the simplest model that captures the main trends in signals acquired from both postmortem and in vivo white matter. We refer to the resulting model of Ref. 1 as the minimal model of white matter diffusion (MMWMD). The MMWMD includes intra- and extra-axonal compartments, similar to Ref. 2, but with a single cylinder diameter rather than the gamma distribution. It also has a cerebrospinal fluid compartment similar to Ref. 3 and a fourth, isotropically restricted, compartment similar to the glial cell compartment of Ref. 5.

Alexander 7 proposed an experiment design optimization algorithm (the active-imaging optimization) that identifies a minimal set of measurements required to be sensitive to the parameters of a model. Simulation experiments demonstrated the potential for orientationally invariant estimation of the axon diameter using a multishell high-angular-diffusion-imaging (HARDI) acquisition with as little as three shells with varying diffusion time, gradient strength, and pulse width. Later work 1 used the active-imaging optimization in combination with the MMWMD to demonstrate orientationally invariant mapping of an index of axon diameter in both a fixed monkey brain and a live human brain. We refer to the technique in Ref. 1 as ActiveAx.

A key compromise in ActiveAx compared to the orientationally specific AxCaliber technique 2,3 is that it uses a single axon diameter parameter rather than the two-parameter gamma distribution model of the axon diameter distribution (ADD). The fitted axon diameter parameter is, thus, a summary statistic of the ADD, which 1 refer to as the “axon diameter index” denoted *a*′. The authors suggest the mean axon diameter weighted by volume, which they call α, as an interpretation of the axon diameter index, *a*′. They, moreover, verified that both statistics discriminate naturally arising ADDs and correlate with one another for distributions that lie within a window of axon diameters to which the imaging protocol is sensitive. This window of sensitivity depends on the set of diffusion MRI data acquired and hence the imaging protocol, which is constrained by hardware limitations as well as the sequence used. In particular, the maximum available gradient strength determines the smallest diameters that can be discriminated; the largest diameters that we can estimate reliably depend on the largest diffusion times we can measure, which in turn depend on the relaxation time constants. The protocol may also affect the level of sensitivity to each axon diameter within the window causing deviations of the axon diameter index, *a*′, from the idealized index, α.

Here, we study experimentally the dependence of the axon diameter index on imaging protocols obtained from the active-imaging optimization using various *G*_max_ and the pulsed-gradient-spin-echo (PGSE) sequence. Diffusion MRI data were acquired ex vivo on a fixed monkey brain using an experimental MR scanner. Axon diameter index maps for various *G*_max_ were calculated and compared. We also repeated the Monte-Carlo simulation experiments in Ref. 1, who used anatomically plausible white matter substrates based on histological data, with each protocol and different levels of signal-to-noise ratio (SNR), to support our findings.

## Methods

### Imaging Protocols

#### Optimization

Optimized imaging protocols based on the PGSE diffusion-weighted (dw) sequence were generated using the active-imaging optimization and a priori axon diameters of 1, 4, and 20 μm. A priori tissue–water diffusivity was set to 0.45 × 10^−9^ m^2^ s^−1^, which is a typical measured apparent diffusion coefficient parallel to the fiber direction in dense white matter at room temperature in our ex vivo sample, and the intracellular fraction was *f*_1_ = 0.7 as in Ref. 1. We included an additional step in the optimization to optimize the number of measurements in each high-angular-diffusion-imaging shell of the number *M* of distinct *b*-values found. Typically, the procedure in Ref. 7 finds only *M* = 3 or 4 unique nonzero *b*-values. For each unique nonzero *b*-value, we assigned a HARDI shell, we added a shell with *b* = 0 s/mm^2^ and initialized each shell with equal numbers of measurements totaling 360. A greedy search algorithm then found, at each step, the reassignment of one measurement to a new shell that gave the largest increase in the objective function. The process terminated when no reassignment improved the score.

For ex vivo imaging, a series of optimized imaging protocols with *G*_max_ constrained to 60, 140, 200, and 300 mT/m was generated. This yielded four protocols, one for each *G*_max_, which we refer to as ActiveAx060, ActiveAx140, etc. The protocols are shown in Table 1.

**Table 1:** Optimized Ex Vivo ActiveAx Protocols Using the PGSE Sequence and Maximal Gradient Strength *G*_max_ of 60, 140, 200, and 300 mT/m

*N*	*G* (mT/m)	δ (ms)	Δ (ms)	*b* (s/mm^2^)	*t*_d_ (ms)	1/*q* (μm)	TE (ms)
*G*_max_ = 60 mT/m							
89	60	15.9	22.4	1121	17.1	24.6	67.2
98	54	15.9	43.3	2032	37.9	27.2	67.2
105	60	26.4	32.9	4312	24.1	14.9	67.2
*G*_max_ = 140 mT/m							
100	140	10.4	16.5	1863	13.2	16.7	49.5
105	108	11.2	29.9	2765	26.4	19.4	49.5
84	140	17.4	23.9	7636	18.1	9.6	49.5
*G*_max_ = 200 mT/m							
102	200	7.7	14.2	1989	11.6	15.2	43.0
106	153	8.8	25.5	2943	22.6	17.4	43.0
81	200	13.8	20.4	8757	15.8	8.4	43.0
*G*_max_ = 300 mT/m							
103	300	5.6	12.1	2081	10.2	13.9	35.9
106	219	7.0	20.4	3080	18.1	15.2	35.9
80	300	10.5	16.9	9542	13.5	7.5	35.9

*t*_d_ = Δ − δ/3 (s), *b*-value = (2π*q*)^2^*t*_d_ (s/m^2^), where *q* = (2π)^−1^γδ*G* (m^−1^) and γ = 2π × 42.57 × 10^6^ (rad/s/T).

The optimization algorithm implicitly optimizes the echo time (TE) in each protocol using an estimate of *T*_2_, which was set to 50 ms. T1 was 800 ms. We also generated for comparison two further optimized imaging protocols with *G*_max_ fixed to 300 mT/m and artificially higher *T*_2_ constants of 400 and 4000 ms (Table 2).

**Table 2:** Optimized Ex Vivo Imaging Protocols Using the PGSE Sequence, Maximal Gradient Strength (*G*_max_) Fixed to 300 mT/m and Artificially Increased *T*_2_ of 400 and 4000 ms

*G* (mT/m)	δ (ms)	Δ (ms)	*b* (s/mm^2^)	*t*_d_ (ms)	1/*q* (μm)
*T*_2_ = 400 ms					
57	11.8	99.1	3035	95.2	35.1
300	7.2	13.7	3765	11.3	10.9
137	9.3	101.6	11,549	98.5	18.4
*T*_2_ = 4000 ms					
3.9	146	200	3452	151.4	41.6
300	7.5	14	4137	11.5	10.5
8.7	146	200	17,280	151.4	18.5

### Subject

One normal young perfusion-fixed Vervet monkey brain (32 months of age) was included in the project and was borrowed for MR scanning from the Montreal Brain Bank 8. The fixation and storage followed the guidelines in Dyrby et al. 9: The brain was perfusion fixated in 4% formaldehyde and postfixed for at least 3 weeks in 1% formaldehyde. The brain tissue was placed in phosphate buffered saline and kept at 5°C for long-term storage. By storing fixed tissue in phosphate buffered saline, free fixative within the tissue is minimized 10. The live monkey was handled and cared for on the Island of St. Kitts according to a protocol approved by the local ethics committee (The Caribbean Primate Center of St. Kitts).

### MRI

Ex vivo diffusion MRI was performed on an experimental 4.7T Varian Imaging System with a bore size of 120 mm and a maximum gradient strength of 400 mT/m with a slew rate of 2000 T/m/s. We used a quadrature volume radio frequency coil and a conventional diffusion weighted PGSE sequence with single-line read out.

#### MRI Setup

To ensure a high quality of the diffusion MRI data set acquired, we followed the guidelines for the preparation and MR scanning stages (III and IV) in Ref. 9: Prior to MR scanning, the temperature of the brain tissue was stabilized to room temperature overnight. The tissue was then sealed in a double plastic bag. Finally, the brain was carefully stabilized in the middle of the volume coil using a mechanically stable setup. During MR scanning, the temperature around the tissue was stabilized by a constant air-conditioned airflow. The temperature of the air flowing out of the magnet was 19 ± 2°C. A diffusion MRI prescan lasting at least 15 h ensured that no short-term instabilities were introduced in the final diffusion MRI dataset [9].

#### Sequence Parameters

All diffusion MRI datasets for the four optimized *G*_max_ protocols (60, 140, 200, and 300 mT/m) shown in Table 1 were acquired in one continuous scanning session lasting about 7 days and repeated 3 months later (selected data is available at http://dig.drcmr.dk). Common scan parameters were isotropic 0.5 mm voxels, no gap between slices, number of excitations = 1, repetition time = 5600 ms, and sagittal slices. Session I included 20 slices for all *G*_max_, whereas session II included 30 slices. For all imaging protocols, the middle slice was aligned with the mid-sagittal plane of the brain. Maximal SNR was ensured by minimizing the TE to 67.2, 49.5, 43.0, and 35.9 ms for *G*_max_ of 60, 140, 200, and 300 mT/m, respectively. All data were visually quality inspected. Visual inspection of acquired diffusion weighted MRI datasets revealed that no within session postprocessing was needed to correct for image motion or artifacts before fitting the data to the tissue model.

### Simulation

#### Synthetic Diffusion MRI Datasets

The Camino 11 Monte-Carlo diffusion simulation system 12 provided synthetic noise-free data from each ActiveAx protocol from various synthetic white matter substrates with known ADD and fiber density. In total, 44 substrates consisting of nonabutting impermeable cylinders with different distribution of diameters and densities were produced as described in detail in Ref. 1. We calculate the idealized axon diameter index, α, for each substrate (Eq. [4] in Ref. 1) from the histogram of cylinder diameters in the substrate. The experiments compare α with the axon diameter index, *a*′, from fitting the MMWMD model. For each substrate and each protocol, we obtain noise-free data from the Monte-Carlo simulation and fit *a*′ 100 times with different independent Rician noise trials with SNR of 20 in the nondiffusion-weighted signal for each protocol. Finally, the experiment was repeated assuming SNR of 20 at TE = 62.7 ms (i.e., for ActiveAx060) and adjusting SNR for the shorter TE of the other protocols assuming *T*_2_ = 50 ms.

Synthesized data were also generated, in a similar way, for a range of single axon diameters: 0.5, 1.0, 1.5, 2.0, 3.0, 4.0, 8.0, and 10 μm but with fixed intracylinder volume fraction of 0.7.

### Fitting the Tissue Model to Diffusion MRI Data

We concentrate here on voxels in which we expect a homogeneous fiber orientation. Thus, we used the same exclusion criteria as in Ref. 1 and fit the MMWMD model using the three-stage fitting procedure outlined therein. The key parameters this estimates are volume fractions of the four compartments in the model and the axon diameter index; diffusivity parameters are fixed as in the original algorithm. The mean of each key parameter was then calculated from its posterior distribution that was constructed using Markov Chain Monte-Carlo in the final stage of the fitting procedure (see Refs.^1^ and 7 for details).

### Analysis

#### ROIs

In this study, we focused on three large and highly dense fiber tracts with different orientations: the corpus callosum (CC), left–right oriented, the corticospinal tract (CST), mostly superior–inferior oriented, and the cingulum (CIN), anterior–posterior oriented. Two sets of regions of interest (ROIs) were drawn, one for each session.

The CC was subdivided into 10 regions as described in Ref. 13. The CC ROIs were manually drawn in the mid-sagittal plane of CC on a non-dw image in session I and then adapted for session II by coregistering non-dw images from each session. For both sessions I and II, the CST and CIN ROIs were manually drawn on the fractional anisotropy (FA) map in each hemisphere. The CST ROIs were drawn on sagittal slices in the pontine region and included about 12 voxels. The CIN ROIs were drawn superior to the splenium region of CC and included about 23 and 12 voxels for sessions I and II, respectively. Within each ROI, the mean axon diameter index and standard deviation of the sample mean was calculated. All ROIs were drawn using the MIPAV program 14, and SPM8 was used for coregistration (http://www.fil.ion.ucl.ac.uk/spm/software/spm).

SNR was calculated as described in Refs.^9^ and 15. The CC ROI was used for the tissue signal, and the background signal was calculated from a ROI drawn in the background of the image.

## Results

### Optimized Protocols

#### Protocol Parameters

The optimized protocols using the PGSE sequence in Table 1 show that the total of 360 measurements consistently distribute as three unique *b*-values (*M* = 3) acquired as HARDI protocols with the number of dw directions (*N*) distributed in the range 80–106 for all shells and about 70 repetitions of *b* = 0 s/mm^2^. The optimized configurations tend to favor more directions at the lowest *b*-value than at the higher *b*-values. In practice, however, the difference in angular resolution using 80 or 106 dw directions is minor.

#### Diffusion Parameters

From Table 1, it can be observed that the diffusion parameters diffusion time, *t*_d_, and the wavenumber, *q*, in the optimized protocols show a weak but systematic trend across *G*_max_: the lowest *b*-value has a short *t*_d_ and a long *1*/*q*, the medial *b*-value has a long *t*_d_ and long *1*/*q*, and the largest *b*-value a long *t*_d_ and short *1*/*q*. Table 2 shows the idealized pattern of large and small *t*_d_ and *1*/*q* combinations that emerges when artificially increasing *T*_2_ to 400 and 4000 ms. Table 1 also shows that δ decreases to keep similar *q*-values as *G*_max_ increases. However, Δ also decreases, so that higher SNR can be expected for higher *G*_max_ as TE decreases. Tables 1 and 2 show how the optimization procedure balances short TE for increasing SNR and long TE to accommodate sufficient diffusion time.

### Simulations

#### Distributed Axon Diameters

**Figure 1:**
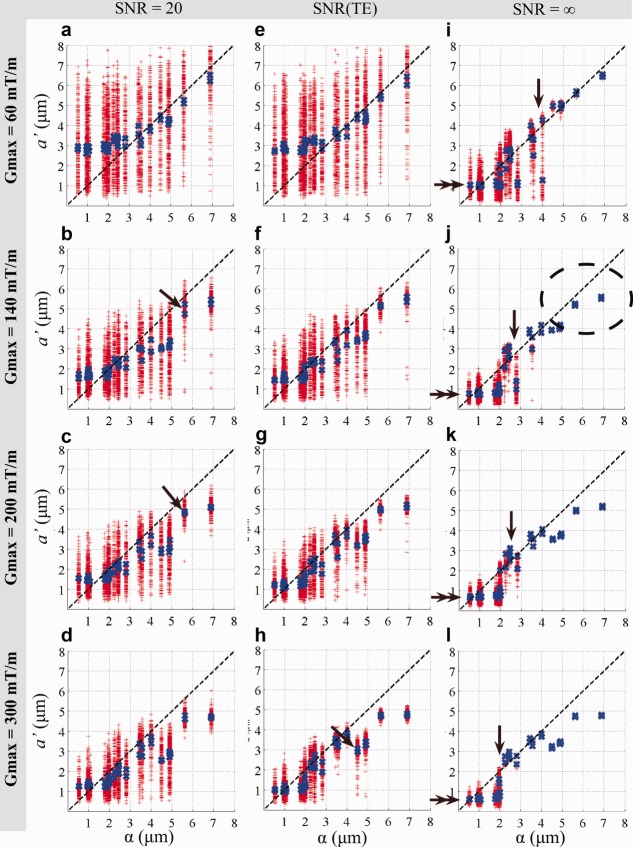
Idealized axon diameter indices α versus estimated indices *a*′ from simulations obtained from different *G*_max_ protocols. The line of identity is indicated by the dashed black line. For *G*_max_ = [60, 120, 140, 300] mT/m, *T*_2_ = 50 ms and (**a–d**) SNR of 20, (**e–h**) SNR adjusted for the different TEs in Table 1, and (**i–l**) noise-free case. For each substrate, the fitting was repeated 100 times (red crosses) and the mean of the repetitions (blue crosses) are shown. The lower bound is visible in the noise-free case at the point where variation of *a*′ appears (i–l, arrow) and the numerical value of *a*′ decreases (i–l, double arrow). The ellipse indicates larger ADDs where the α overestimates *a*′.

The variation of the mean axon diameter index estimated from the 100 repeated experiments on the 44 synthetic substrates with fixed SNR of 20 decreases with increasing *G*_max_ as shown in Figure 1a–d. Accounting for increasing SNR as TE is reduced (Fig. 1e–h) emphasizes the effect. However, the effect of *G*_max_ is much stronger than SNR. In both cases, variation for a *G*_max_ of 140 mT/m and higher, gradually decreases as *G*_max_ increases (Fig. 1a–h). In general, the variation of *a*′ decreases, as *a*′ and α increase. This effect is clearly seen in the noise free case where variation of *a*′ appears only beneath a lower bound on the window of sensitivity (Fig. 1i–l, arrows). The mean axon diameter index over the 100 trials (blue crosses) is consistent for substrates with the same ADD but with different fiber density and is observed as overlapping blue crosses (Fig. 1b–h, arrow).

**Figure 2:**
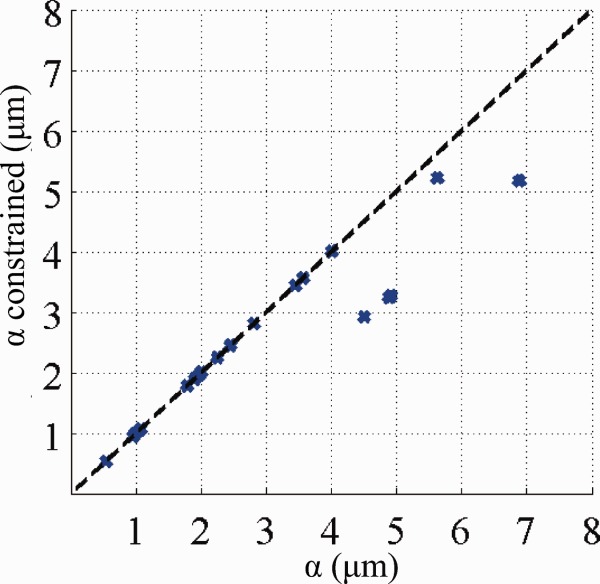
Idealized axon diameter indices α with and without the exclusion of large axon diameters (>10 μm).

**Figure 3:**
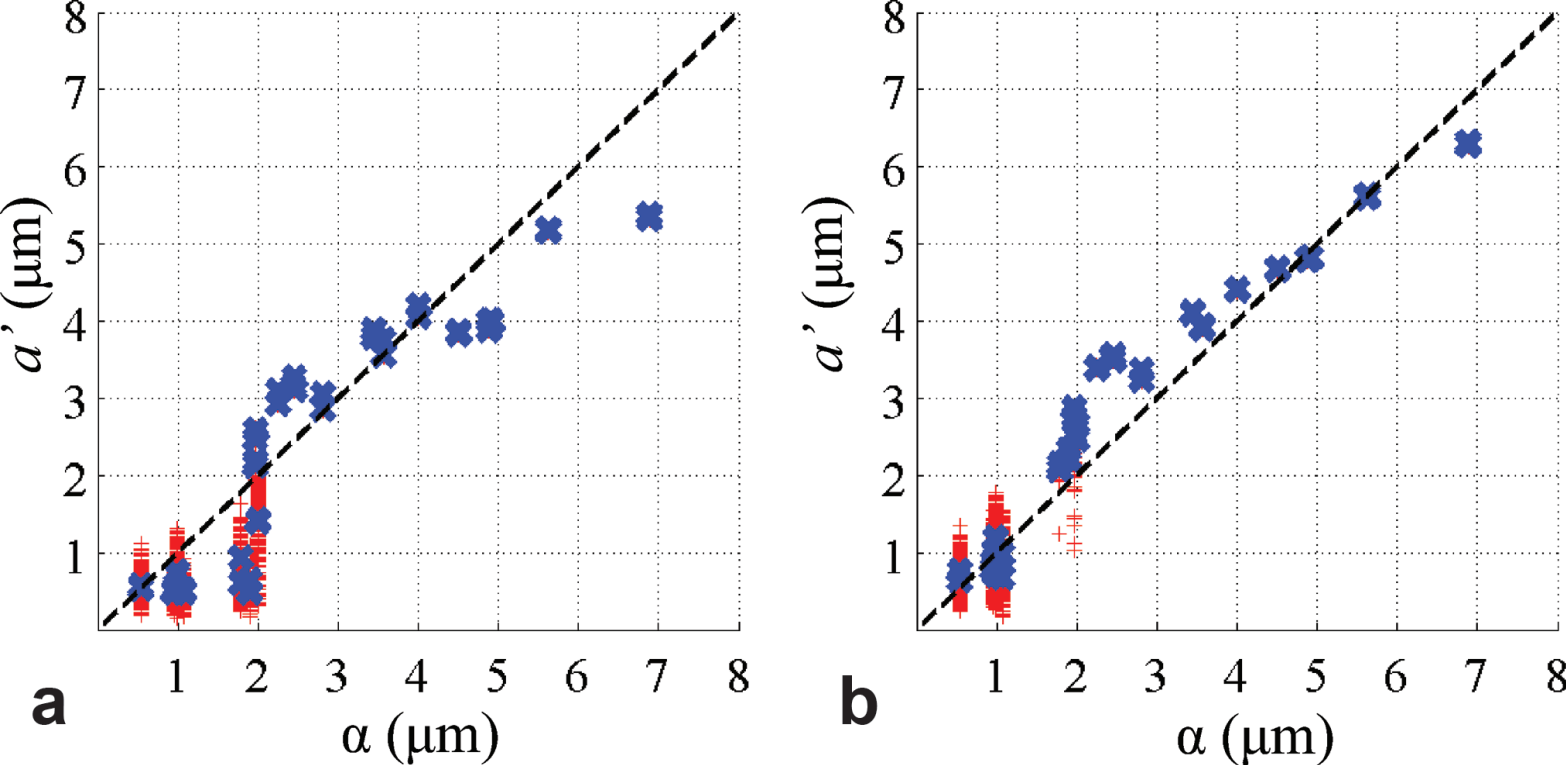
The effect of *T*_2_ on the axon diameter index. Simulation results on ADDs for ActiveAx300 in a noise-free case when *T*_2_ is increased from 50 ms in Figure 1l to (**a**) 400 ms and (**b**) 4000 ms.

The mean axon diameter index generally reflects the idealized index, α, but systematic differences arise. First, the noise-free experiment clearly identifies a “lower bound” (Fig. 1i–l, arrows) on α below which *a*′ shows a large variation (red crosses). With the addition of noise the lower bound cannot be identified from variation but is still apparent from the mean values of *a*′ (blue crosses). Both the lower bound (Fig. 1i–l, single arrows, blue crosses) and the numerical values of mean *a*′ below the lower bound (Fig. 1i–l, double arrows, blue crosses) decrease, as *G*_max_ increases. Second, *a*′ departs from α as α gets larger, and the effect becomes more pronounced as *G*_max_ increases, as indicated in Figure 1J, dashed ellipse. The interpretation of *a*′ as α breaks down when the distribution of diameters includes very large values (>10 μm). These large diameters have a strong effect on α but are out of range of the diffusion times in the ActiveAx protocols and therefore do not contribute to *a*′. Figure 2 plots for all 44 substrates α as in Figure 1 against α recalculated when ignoring all diameters above 10 μm; the recalculated α shows a similar pattern to *a*′ in Figure 1. On the other hand, Figure 3 shows that the protocols optimized with artificially higher *T*_2_ (Table 2), which include much longer diffusion times, provide an *a*′ that is sensitive to the larger diameters.

#### Single Axon Diameter Estimation

**Figure 4:**
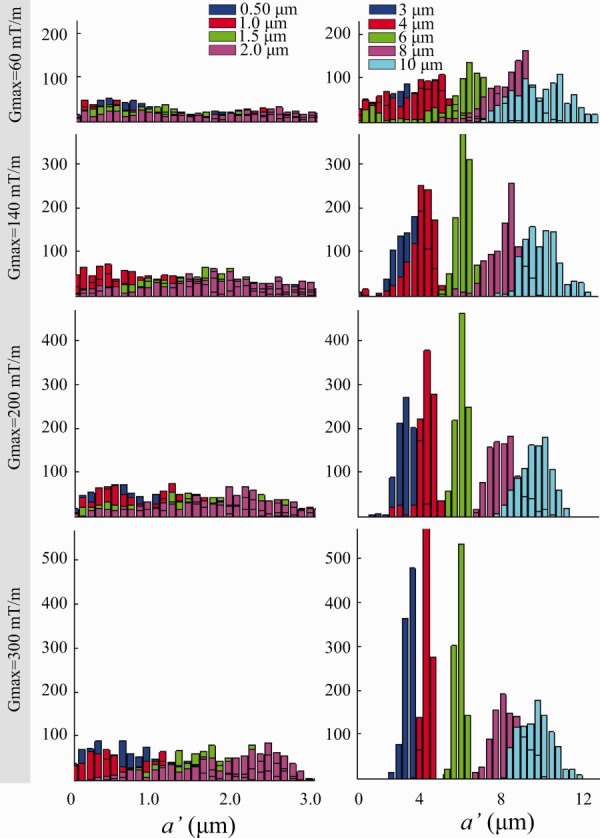
Posterior distributions on *a*′ for single axon diameter synthetic data. For *G*_max_ = 60, 140, 200, and 300 mT/m, posterior distributions for axons with diameters of 0.5, 1, 1.5, and 2 μm and 3, 4, 6, 8, and 10 μm are shown in the left and right columns, respectively.

Figure 4 shows posterior distributions of *a*′ from various sets of data synthesized from single axon diameters rather than the distributions used for Figure 1. This allows us to investigate the sensitivity of ActiveAx for very narrow distributions without the smearing effect from heterogeneous ADDs. In combination, the histograms illustrate more directly the window of sensitivity and its dependence on *G*_max_. Single axon diameters smaller than 3 μm have broad and overlapping posterior distributions for any *G*_max_ and support the lower bounds observed in Figure 1. For any *G*_max_, Figure 4 shows little contrast between posterior distributions below the lower bound, and the noise-trial results in Figure 1 suggest that we cannot expect higher SNR to improve contrast. For larger axon diameters, the distributions become narrower and separated especially for higher *G*_max_. However, particularly for the highest *G*_max_, the variance of the posterior distribution of the largest diameters (e.g., 10 μm at *G*_max_ = 300 mT/m) becomes noticeably broader than that for the mid-range diameters (e.g., 4 μm). This suggests that, although larger axons do influence the signal enough to recover their diameter, the relatively short diffusion times cause fewer spins to experience the effect of a boundary, and the signal-contrast is weaker than for smaller axons diameters. This same effect causes the deviation of *a*′ from α that we observe for the data points in Figure 1 corresponding to ADDs including diameters over 10 μm. The definition of α assumes uniform sensitivity across all axon diameters, whereas our measurements, and thus *a*′, are preferentially sensitive to mid-range axon diameters.

### Monkey Data

#### CC

The SNR measured in CC for sessions I and II is [18, 24, 28, 32] and [19, 26, 29, 35] for *G*_max_ of 60, 140, 200, and 300 mT/m, respectively, thus SNR increases as expected with higher *G*_max_ as TE decreases.

**Figure 5:**
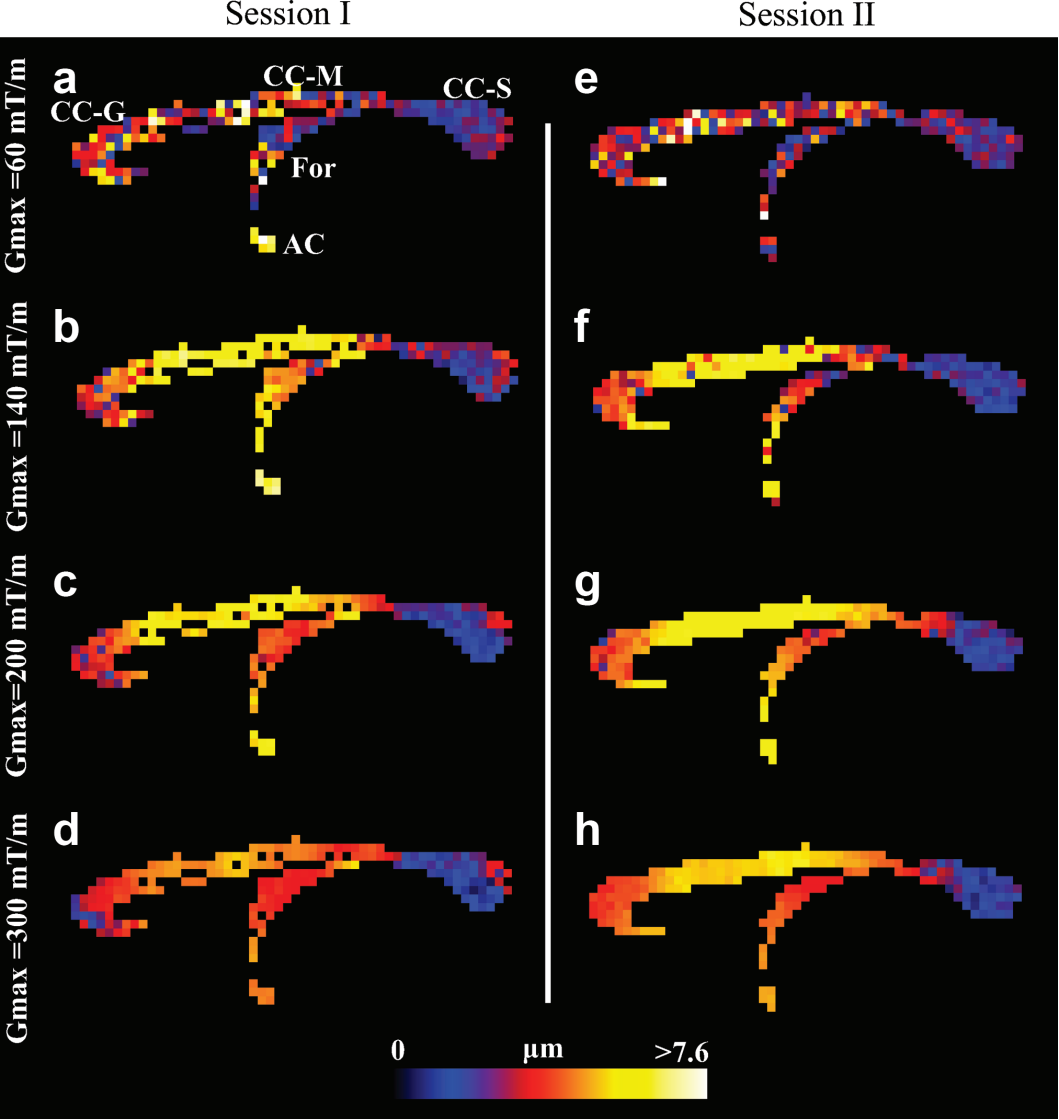
Voxel-wise estimation of axon diameter index in the CC of the fixed monkey brain. Axon diameter index obtained from sessions I (**a**–**d**) and II (**e**–**h**), shown in a midsagittal slice for *G*_max_ = 60, 140, 200, and 300 mT/m. The range of diameters is shown by the color bar. High agreement between the two sessions is observed. Higher *G*_max_ improves spatial coherence and more anatomical details appear. Abbreviations: Genu (CC-G), midbody (CC-M), and splenium (CC-S) regions of CC, fornix (For), and anterior commissure (AC).

The voxel-wise axon diameter indices in the midsagittal plane of CC for all *G*_max_ shown in Figure 5 clearly get less noisy with higher *G*_max_. Regional clusters of different axon diameter indices, especially in splenium, fornix, and anterior commissure, become clear as *G*_max_ increases, especially for *G*_max_ > 140 mT/m. The simulations in Figure 1 predict a reduction in variation of *a*′ at higher *G*_max_, and it is clearly seen from Figure 5 how this enhances the anatomical detail visible in the axon diameter index map.

A small amount of Gibbs ringing occurred in the *b* = 0 s/mm^2^ image in session I, which prevents successful fitting of the model in a few CC voxels; the figure shows those voxels as zero diameter (black), like the background.

**Figure 6:**
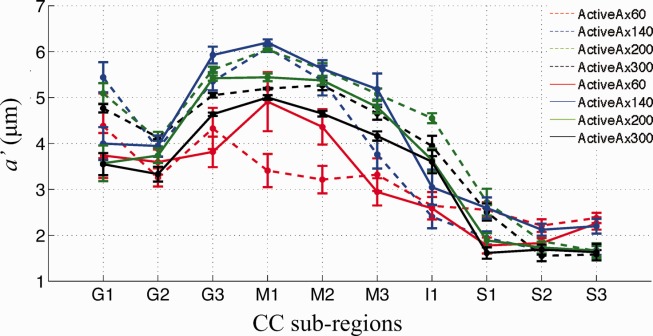
Mean and standard error of *a*′ for various *G*_max_ within 10 subregions in CC. Sessions I and II are shown as solid and dashed lines, respectively. Similar trends in *a*′ across CC subregions are seen across *G*_max_. Generally, the axon diameter index and its variance both decrease as *G*_max_ increases.

Mean axon diameter index in CC subregions follows a similar and clear low–high–low trend for all *G*_max_ settings (Fig. 6). Generally, *G*_max_ of 60 mT/m produces the lowest mean diameter index across most CC subregions, followed by a *G*_max_ of 300, 200, and 140 mT/m. For all *G*_max_, the variation of the index (error bars in Fig. 6) in each CC subregion is minor especially for *G*_max_ > 60 mT/m but tends to be smaller where the index itself is larger, for example, in the CC mid-body, as the simulations predict. The largest variation is seen in G1, which is the region that includes the most anterior tip of genu and thus has increased likelihood of partial volume contamination.

Differences in the axon diameter index between sessions are generally larger than within-ROI variation. This may arise from the Gibbs ringing artifacts, residual misalignment of the two CC ROIs, or additional smoothing introduced by the alignment of session I to session II.

#### CST and CIN ROI Analysis

**Figure 7:**
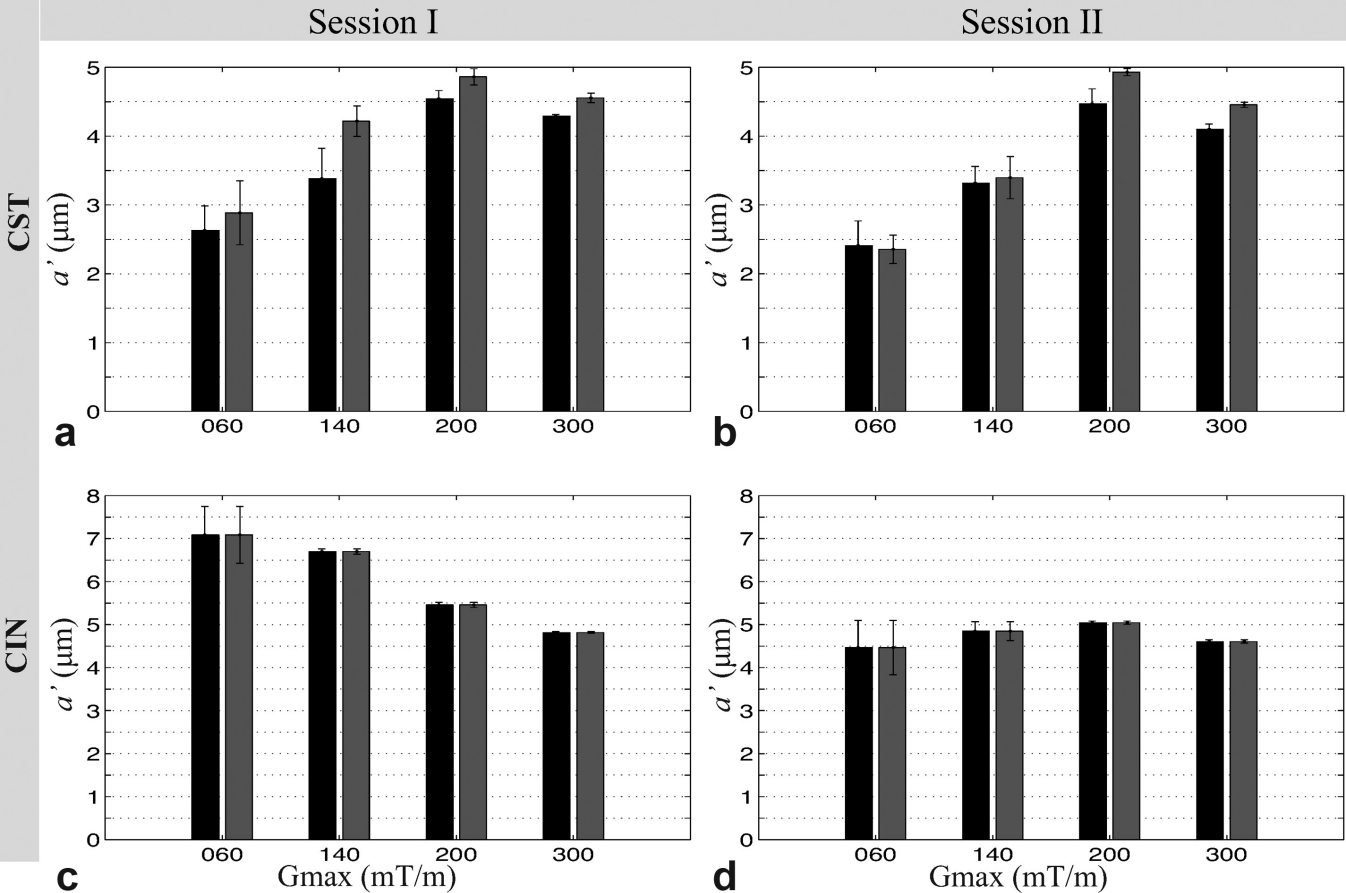
Mean and standard error of *a*′ within a region of the CST and CIN of the fixed monkey brain. Results for the left and right hemispheres are shown as black and gray, respectively, for both scanning sessions.

For any *G*_max_, high consistency was found in mean axon diameter index between the right and left side of CST and CIN for both sessions I and II, as shown in Figure 7. No statistical difference in axon diameter index was found between the right and left side of CST and CIN. No image artifacts are apparent in the CST and CIN regions. The data sets with *G*_max_ larger than 140 mT/m provide consistent estimates of axon diameter index in CST and CIN ROIs, and the between-session differences are less than the within-ROI variation.

## Discussion

We show by combining simulations on microstructural substrates and lengthy ex vivo scanning sessions, normally not practically possible, how the available gradient strength (*G*_max_) plays a key-role in providing useful contrast in diffusion based axon diameter index maps. Our results provide new insights into the performance of current microstructure imaging techniques. Although we have used the ActiveAx technique 1 throughout, our findings are also relevant for the range of similar microstructure imaging techniques 2–5,16,17. Taking those into account is pivotal for the future translation of these promising techniques into clinical practice.

### Optimized Protocols

The optimized ActiveAx protocols all include three unique *b*-values as expected from Refs.^7^ and 1 (the protocol in the latter includes four shells, but two are almost identical and could be merged). The *t*_d_ and *q*-values that define each shell were comparable across *G*_max_, especially for the higher *G*_max_, which was expected, because all ActiveAx protocols were optimized for the same a priori model parameter settings.

### Variation of Axon Diameter Index

The variation of the axon diameter index for the lowest *G*_max_ of 60 mT/m was significantly greater than for any higher *G*_max_. One key reason for this is the wider gradient pulses (δ) needed to obtain sufficient diffusion weighting (high *q*-value) at this low *G*_max_. This increases TE and thus reduces SNR. However, SNR, which can be increased in practice by repeating measurements or increasing voxel size, is not the most important parameter reducing performance at *G*_max_ = 60 mT/m compared to higher *G*_max_. A more fundamental limitation is that long pulses prevent short diffusion times thereby reducing sensitivity to smaller axon diameters, reducing anatomical contrast.

Our results suggest always using the highest possible *G*_max_, at least up to the maximum used here (300 mT/m). It seems likely that even higher *G*_max_ will provide further details. The results suggest using a *G*_max_ of at least 140 mT/m whenever possible, as at this gradient strength many anatomical details that are not discernible at lower *G*_max_ will then appear. However, even the lowest *G*_max_ of 60 mT/m (currently achievable on some clinical systems) still produces useful contrast, providing unique and specific microstructural differences that are not extractable from diffusion tensor imaging (DTI).

The simulations in Figure 1 show that, even with the highest *G*_max_ of 300 mT/m, it can be hard to distinguish, using *a*′, a particular ADD with α = 3 μm from a particular distribution with α = 5 μm. However, the ADDs used in Figure 1 are only representative distributions for demonstrating how practical constraints such as *T*_2_, *G*_max_, and SNR impact upon *a*′. A wide range of distributions can have any particular value of α. Other pairs of distributions with α = 3 and 5 μm are distinguishable; the idealized delta distributions (single axon diameters) in Figure 4 provide an example, as the posterior distributions on *a*′ for 3 and 5 μm clearly separate. In combination, those results suggest that the width of the ADD plays an important role in the contrast we get from *a*′: the tighter the distributions, the more discriminating *a*′. The relatively large variation of *a*′ in Figure 1 (red crosses) compared to the smooth variation in brain data (Fig. 5) suggests that the underlying ADDs in tissue are probably narrower than the simulated situation. Further work to perform the painstaking electron microscopic (EM) analysis of CC subregions in the Vervet monkey brain combined with further simulations is needed to support this suggestion.

The variation of the axon diameter index across *G*_max_ stresses the fact that it is not a fully quantitative measurement. However, the contrast is important even if the absolute values are harder to interpret. The axon diameter index map may be considered an axon-diameter-weighted image rather than a quantitative map of mean axon diameter. Nevertheless, the contrast is a unique and useful tool for studying brain anatomy nondestructively as, for example, the axon diameter index captures the same broad trends in axon size as reported in histological studies 13,18,19. The axon diameter index, therefore, provides unique information about conduction velocity in white matter and, thus, brain function.

### Sensitivity to Axon Diameter

Simulations provide unique insight into the window of sensitivity of ActiveAx and other axon diameter imaging techniques that use PGSE. In particular, (i) the existence of a lower bound of axon diameters below which small axon diameters are identified as small but cannot be distinguished from one another; and (ii) the suggestion of an upper bound above which sensitivity is reduced because of insufficient diffusion time.

When the ADD is entirely below this upper bound, α usually provides a good interpretation of *a*′, although a wide ADD may also cause departures owing to variation in sensitivity across its support. However, the most significant departures occur when the distribution extends above the upper bound, α is larger than *a*′, because larger axons have a stronger influence on α than they have on *a*′ due to the relative short diffusion time.

The simulation experiments with truncated distributions of axon diameters (Fig. 2) highlight the upper bound on the axon diameter index deliberately by excluding portions of distributions with very large axons (>10 μm). However, the single diameter simulations in Figure 4 reveal a more subtle behavior. Even for the ActiveAx300 protocol, where the largest diffusion time is only 18 ms (root mean squared displacement of around 4 μm), the sensitivity of the measurements to 10 μm diameters is sufficient to estimate the diameter (22% of spins starting in the center of a 10 μm axon still reach the boundary). However, the variance of the posterior distribution for the 10 μm diameter axons (cyan histogram in Fig. 4 row 4) is higher than that for lower diameters, suggesting weaker (but nonzero) sensitivity for the larger axon diameters. We can obtain an interpretation of *a*′ for any ADD that is more accurate than α by including a modulation by a sensitivity function *f* over axon diameters in the original expression for α (Eq. [4] in Ref. 1):

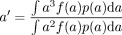
where *p* is the distribution of axon diameters, and ∫ *f* (*a*) da = ∫ *p* (*a*) da = 1. For α, *f* is constant. For *a*′, *f*(*a*) appears to approximate the variance of the posterior distribution at *a*. The results for ActiveAx060 in Figure 4 support this interpretation, as the variances are more consistent, albeit larger, across mid, and large range diameters than they are for ActiveAx300; correspondingly, at large α, α is closer to *a*′ in Figure 1 for ActiveAx060 than for ActiveAx300. However, any link between the variance of the posterior distribution and the sensitivity function remains speculation until further work can test its validity.

Sensitivity to large diameters is limited by the *T*_2_ of the sample, which can be significantly reduced (30–50 ms) for fixed brain tissue. If we ignore the effects of *T*_2_, as in Figure 3, the active-imaging optimization includes measurements with long diffusion times that enhance sensitivity at the largest a priori diameters that the optimization considers (20 μm). However, those measurements require very long TE and give no signal when *T*_2_ is low.

If equal sensitivity across the range of diameters is important, one might adapt the optimization from Ref. 7 to equalize expected variance at each a priori diameter. However, this will as seen for ActiveAx60 sacrifice contrast to the most sensitive part of the range for consistency with less sensitive parts reducing overall contrast among ADDs. We believe the current approach is desirable, as the effect of the optimization is to maximize contrast between the axon diameters in the most sensitive range.

In practice, axons with diameter over 10 μm are rare, so the values of *a*′ in the monkey brain data are likely to match α. Should we need to increase sensitivity to larger axons, or if *T*_2_ is even lower, a stimulated echo sequence is expected to be beneficial, as it enables long diffusion times with short TE. Indeed, some earlier methods 2,5 use stimulated echo for precisely this reason. Here, we focus on PGSE, because it is easier to achieve for in vivo and clinical imaging 1,3, but future work will investigate the impact of including long diffusion times via stimulated echo.

Simulations elucidated the existence of a lower bound of a few micrometers below which axon diameters are identified as small but not discriminated. The lower bound is a fundamental consequence of limited *G*_max_ rather than an artifact of the optimization algorithms; note that we included in the optimization of the imaging protocols an a priori axon diameter of 1 μm, which is below all lower bounds. The lower bound arises, because with small axons, the largest possible internal perpendicular displacements are not large enough to cause detectable signal attenuation for any configuration of the PGSE sequence with limited *G*_max_. As the gradient strength increases, PGSE configurations can be found that produce signal attenuation for smaller axons, because we can combine higher diffusion weighting with shorter diffusion times. However, the lower bound decreases only slowly as *G*_max_ increases. Replacing the rectangular pulses with switching or oscillating gradient waveforms enables diffusion weighting with shorter effective diffusion times 20. These alternative gradient waveforms produce detectable signal attenuation for smaller displacements and thus potentially reduce the lower bound compared to PGSE for fixed *G*_max_. Recently, Drobnjak et al. 21 generalized the active-imaging optimization to search the much wider measurement space with arbitrary gradient waveforms instead of the rectangular pulses in PGSE. Their simulations showed that the optimal gradient waveforms for sensitivity to small axon diameters include switching gradients, and as the axon diameter gets smaller, a higher switching frequency is needed. Future implementation of these ideas promises sensitivity over a greater portion of the range of naturally occurring diameters and even greater anatomical contrast.

### Translating Ex Vivo Into In Vivo

Here, we assumed that the microstructural environment and its length scales and restrictions are preserved ex vivo 22, which is supported by diffusion tensor studies showing that anisotropy is preserved ex vivo 23. Due to lower temperature and cross-binding of proteins caused by the fixative 24, diffusivity in fixed tissue is around three times lower than in vivo, and *T*_2_ is also typically reduced 10. These few basic differences between the ex vivo and in vivo environment allow some speculation on how our conclusions on microstructural imaging can be extrapolated to the in vivo situation. The upper bound on the axon diameter sensitivity will be higher due to the higher diffusivity and *T*_2_ in vivo. However, the lower bound for correctly detecting small axons is likely to be similar in vivo and ex vivo for the same *G*_max_. The importance of a high *G*_max_ to ensure accuracy and minimal variation of estimation to microstructural details in vivo applies to all scanners (clinical or preclinical) independent of field strength. We hope that the major improvements we demonstrate from increasing *G*_max_ will help expedite the development of stronger gradient systems for commercial human scanners.

## Conclusions

The influence of the available gradient field strength on estimated axon diameter indices was investigated for the first time. Fixed monkey brain results show clear improvements in regional contrast in axon diameter index maps as *G*_max_ increases, whereas higher SNR is less important. We conclude that a *G*_max_ of 300 mT/m reveals anatomical details not seen at lower *G*_max_ values (even with higher SNR), and one should, therefore, use the highest possible *G*_max_ in these types of studies.
